# Prospective study on physical activity and Alzheimer's disease–related biomarkers

**DOI:** 10.1002/alz.71755

**Published:** 2026-08-19

**Authors:** Paula Iso‐Markku, Xin M. Tu, Robert A. Rissman, Nathan A. Gillespie, Jeremy A. Elman, Christine Fennema‐Notestine, Matthew S. Panizzon, Chandra A. Reynolds, Michael J. Lyons, William S. Kremen, Carol E. Franz, for the Vietnam Era Twin Study of Aging

**Affiliations:** ^1^ Department of Psychiatry University of California San Diego, La Jolla California USA; ^2^ Center for Behavior Genetics of Aging University of California San Diego, La Jolla California USA; ^3^ Institute for Molecular Medicine Finland (FIMM), HiLIFE University of Helsinki Helsinki Finland; ^4^ Clinical Physiology and Nuclear Medicine HUS Diagnostic Center University of Helsinki and Helsinki University Hospital Helsinki Finland; ^5^ Institute for Behavioral Genetics University of Colorado Boulder Colorado USA; ^6^ Department of Psychology & Neuroscience University of Colorado Boulder Colorado USA; ^7^ Virginia Institute for Psychiatric and Behavior Genetics Virginia Commonwealth University Richmond Virginia USA; ^8^ Department of Psychology Boston University Boston Massachusetts USA

**Keywords:** Alzheimer's disease, amyloid, biomarkers, cognition, neuroinflammation, Physical activity, tau

## Abstract

**INTRODUCTION:**

Evidence regarding the association between physical activity (PA) and Alzheimer's disease (AD)–related biomarkers is scarce and inconsistent.

**METHODS:**

At ages 56 and 67, 564 men from the Vietnam Era Twin Study of Aging reported their PA during the preceding week from which metabolic equivalent of energy expenditure (MET) hours were determined. At age 67 we assayed plasma AD‐related biomarkers (phosphorylated tau [p‐tau]217, neurofilament light chain [NfL], amyloid beta (Aβ)42/40 ratio, and glial fibrillary acidic protein [GFAP]). We used generalized estimating equations to test whether PA at ages 56 or 67 was associated with these biomarkers at 67.

**RESULTS:**

Age 56 MET hours were inversely associated with NfL and GFAP at 67 (NfL: B = −0.10, 95% confidence interval [CI]: −0.17 to −0.03, GFAP: *B* = −0.08, 95% CI: −0.15 to −0.01) but were not significantly associated with p‐tau217 or Aβ42/40 ratio.

**DISCUSSION:**

Our NfL and GFAP results suggest that midlife PA may reduce risk for neurodegeneration in older age via amyloid‐independent mechanisms.

## Highlights

- Midlife physical activity (PA) was inversely associated with early late‐life blood‐based neurofilament light chain and glial fibrillary acidic protein.
- Moderate‐to‐vigorous, but not light, PA appeared to account for the association between midlife PA and later biomarkers.
- PA may be protective during aging by reducing neurodegeneration and inflammation.

## 1 BACKGROUND

Physical activity (PA) has been associated with reduced risk for dementia.^1^ Although evidence from animal studies suggests that PA decreases amyloid and tau accumulation and protects against neuroinflammation,^2^, ^3^, ^4^, ^5^ Alzheimer's disease (AD)–related biomarker evidence from human studies is scarce and inconsistent.^6^ The discovery of blood‐based biomarkers for AD has revolutionized the diagnostics and research of AD. Blood phosphorylated tau (p‐tau)217 is a reliable blood‐based biomarker of AD pathology, correlating with both hallmarks of AD: cerebral amyloid and tau accumulation.^7^, ^8^ Low blood amyloid beta (Aβ)42/40 ratio also reflects high cerebral amyloid accumulation^9^. Blood glial fibrillary acidic protein (GFAP) is thought to be a marker of neuroinflammation and astrocytic activation^10^ and neurofilament light chain (NfL) is an unspecific marker of neurodegeneration, with especially elevated levels seen in the presence of white‐matter alterations^11^ and in small‐vessel disease.^12^

In transgenic rodents, it has been shown that voluntary exercise reduces Aβ in the brain and prevents amyloid^2^ and hyperphosphorylated tau accumulation.^2^, ^3^ Aerobic exercise in young rodents seems to target primarily amyloidogenic amyloid precursor protein processing and Aβ degradation while long‐term running in older rodents with advanced stages of AD affects Aβ clearance across the blood–brain barrier, autophagy machinery, anti‐inflammatory processes, and anti‐oxidative effects.^4^ Running has also been shown to increase hippocampal astrocyte plasticity in rodents.^5^ To our knowledge, the association between aerobic exercise and the levels of NfL has not been studied in animal models.

In humans, most of the evidence on the association between exercise and Aβ levels is from observational cross‐sectional studies in mainly adults older than 65 years using enzyme‐linked immunosorbent assay (ELISA) techniques to detect Aβ in cerebrospinal fluid (CSF) or blood.^6^ To our knowledge, most earlier studies on PA and blood biomarkers of amyloid accumulation have used less sensitive methods to detect amyloid accumulation (Aβ38, Aβ40, Aβ42, or Aβ42/40 detected with ELISA), and have found inconsistent results (no significant association, lower or elevated levels of Aβ38, Aβ40, Aβ42, or Aβ42/40)^6^. Results from longitudinal studies of PA are inconsistent. Two large longitudinal studies on PA and blood‐based Aβ^13^, ^14^ together with one longitudinal study on PA and positron emission tomography (PET)–measured Aβ^15^ show no association while the single largest longitudinal study of PA and PET‐measured cerebral amyloid shows an inverse association.^16^ A meta‐analysis of randomized controlled trials examining the association between exercise and Aβ‐related pathology showed no effect of exercise on amyloid metabolism in either brain, CSF, or blood.^17^

The association between exercise and tau pathology seems to tell a similar story: There is no convincing evidence that physical exercise is associated with CSF‐ or PET‐measured total tau or CSF p‐tau^18^, ^19^ or with plasma p‐tau181^13^, ^20^, ^21^, ^22^, ^23^ in adults older than 65 years. An exception would be the study by Yau et al.^15^ in which they found that > 80% of the longitudinal association between PA and slower cognitive decline was mediated by cerebral tau burden in adults on average 70 years old.

Findings on the association between PA and NfL levels are scarce, with most studies showing no association.^13^, ^23^, ^24^ A few cross‐sectional studies showed an inverse association^25^ or an inverse association with a saturation effect meaning that the inverse association was only seen with up to 5 hours of light PA or 2 hours of moderate‐to‐vigorous PA but not at higher PA levels.^26^ The scarce human studies on the association between physical exercise and plasma GFAP show either no association^13^ or an inverse association.^23^, ^27^ These findings are of particular interest because PA is considered a modifiable risk factor for dementia, yet the nature of this relationship remains unclear.^28^

To sum up, earlier studies on the association of PA and AD‐related blood biomarkers are mostly cross‐sectional and use the less sensitive ELISA‐based techniques to detect AD‐related blood biomarkers. Additionally, there is a discordance between human‐ and animal‐based evidence on the association between PA and AD‐related pathology. It is possible that PA is associated with healthier cognitive aging via different mechanistic pathways in humans and in animals. For example, in animals, amyloid‐ and tau‐dependent mechanisms may prevail, while, in humans, decreased neuroinflammation, decreased cerebrovascular pathology, more effective mitochondrial capacity, and improved synaptic plasticity may dominate^29^. In the present prospective study, we examined the association of mid‐ and early late‐life self‐reported PA with early late‐life blood‐based Aβ42/40 ratio, p‐tau217, GFAP, and NfL using ultrasensitive immunoassays. This study can provide insight into mechanisms linking PA with cognitively healthier aging and the findings can aid in earlier identification of individuals on a dementia trajectory allowing for potential early interventions.

## 2 METHODS

### 2.1 Study population

**RESEARCH IN CONTEXT**

1. **Systematic review**: We reviewed the literature using traditional (e.g., PubMed) sources and found a discrepancy in mechanisms by which physical activity (PA) protects against cognitive decline. In Alzheimer's disease (AD) rodent models, PA decreases both amyloid and tau deposition but evidence of a similar strong association from human studies is lacking. Additionally, although PA decreases chronic systemic inflammation, direct evidence of protection against neuroinflammation remains feeble.
2. **Interpretation**: Our findings suggest that midlife PA, in particular, moderate‐to‐vigorous PA, protects against cognitive decline primarily via amyloid‐ and tau‐independent mechanisms, possibly via reducing cerebrovascular pathology and neuroinflammation.
3. **Future directions**: Future studies should explore (a) the potentially biphasic patterns of AD‐related biomarkers during AD course and (b) how PA affects these processes during different phases of the AD course using longitudinal study designs, fine measures of PA, and ultrasensitive biomarkers or in vivo imaging of AD neuropathology.

The Vietnam Era Twin Study of Aging (VETSA) is an ongoing study of cognitive aging and early identification of risk for dementia.^30^ The study cohort is part of the Vietnam Era Twin (VET) Registry, which comprises male twin pairs who served in the military at some time during Vietnam era (1965–1975).^31^ VETSA participants were randomly selected from participants of a previous study using VET Registry members.^32^ They participated in thorough examinations comprising broad health questionnaires, functional assessments, and extensive cognitive testing in three waves. The present study consists of 564 initially cognitively unimpaired study participants who completed their baseline PA assessment at VETSA wave 1 between 51 and 61 years of age (mean age 55.9, standard deviation [SD] 2.4) and had data on AD‐related blood biomarkers from VETSA wave 3 (mean age 67.3, SD 2.6). Wave 1 data are referred to from hereon as “age 56” and wave 3 data are referred from hereon as “age 67.” Participants gave written informed consent to participate. The study was approved by institutional review boards at University of California, San Diego and Boston University. Participants could choose to go to either site where identical protocols were administered. The study was performed in accordance with the ethical standards as laid down in the 1964 Declaration of Helsinki and its later amendments.

### 2.2 Cognitive status

Our diagnosis of mild cognitive impairment (MCI) has been explained extensively elsewhere^33^, ^34^. It was based on 18 neuropsychological measures covering episodic memory, executive function, attention/working memory, verbal/language, visual‐spatial, and processing speed domains. Using the Jak–Bondi approach,^35^ impairment was defined as having ≥ 2 tests in a domain that were > 1.5 SD below external normative means. Individuals who did not meet impairment criteria in any domain were classified as cognitively unimpaired. Study participants with MCI at wave 1 were excluded (*n* = 70).

### 2.3 Physical activity

At ages 56 and 67, study participants were asked to list any sports, fitness, or recreational PA in which they participated during the preceding week (see Supplement in supporting information).^36^ In addition to the type of PA, they were also asked to report the frequency and duration of these activities (see Supplement). The activities were categorized into light, moderate, and vigorous activities according to classification of energy costs of human physical activities by Ainsworth et al.^37^ During training, staff had to reach reliability levels > 95% prior to using the coding system. The World Health Organization (WHO) defines light PA as PA at an intensity of 1.5 to 3 times metabolic energy expenditure at rest (metabolic equivalent of task [MET]), moderate PA as PA at an intensity of 3 to 5.9 MET, and vigorous PA as PA at an intensity of at least 6 MET.^38^ To estimate MET hours we used the averages of light and moderate PA MET ranges as approximations in our calculations for light and moderate intensity PA (light = 2.3 MET, moderate = 4.5 MET) while for vigorous PA, we used the approximation of 8 METs similar to as in the International Physical Activity questionnaire^39^. We then multiplied intensity by frequency and duration (intensity x frequency x duration)^34^ yielding the mean amount of PA during the preceding week for each study participant. In addition to MET hours, we used a binary (yes = 1/no = 0) variable (WHO‐PA) describing whether the study participants met the WHO‐PA recommendation (150 minutes of moderate PA or 75 minutes of vigorous PA or a combination per week).^38^ This variable was based on continuous MET hours described above.

### 2.4 Plasma biomarkers

Plasma was collected under fasting conditions at age 67.^40^ For the Aβ42/40 ratio, the Simoa Human Neurology 3‐plex A (N3PA) Quanterix HD‐X platform was used.^41^ For NfL, GFAP, and p‐tau217, the Simoa technology platform HD‐X was used.^42^ All assays were performed according to manufacturer's instructions. Details of the procedures are published elsewhere.^40^ Participants with values outside 3 SD from the mean (5 for NfL, 1 for p‐tau217, 19 for GFAP, 18 for Aβ42, and 28 for Aβ40) or with coefficient of variation > 0.20 (2 for NfL, 2 for p‐tau217, 0 for GFAP, 10 for Aβ42, and 5 for Aβ40) or hemolyzed samples (37 out of 1108) were removed before the analyses.

### 2.5 Covariates

The covariates used in this study were age, race/ethnicity (non‐Hispanic White vs. other), body mass index (BMI), socioeconomic status, smoking, alcohol consumption, apolipoprotein E (*APOE*) genotype, multimorbidity index, and glomerular filtration rate (GFR). For analyses of age 56 PA predicting age 67 biomarkers, age and measures of BMI, socioeconomic status, smoking, alcohol consumption, and multimorbidity were from age 56 as well as age 67 GFR. In cross‐sectional analyses at age 67, age, PA status, BMI, smoking, alcohol consumption, multimorbidity, and GFR were from age 67. BMI was calculated from in‐person measurements. Socioeconomic status was based on the Hollingshead–Redlich index (5 x average occupation on a 1–9 scale + 3 x average education on a 1–7 scale)^43^. Smoking was used as a dichotomous variable (current smoker vs. non‐smoker). Alcohol consumption was used as a continuous variable describing the self‐reported number of drinks during the previous 2 weeks. *APOE* genotype was used as a dichotomous variable (no ε4 allele vs. at least one ε4 allele). *APOE* isoform (ε2, ε3, and ε4) was determined from genome‐wide genotyping using the single‐nucleotide polymorphisms rs429358 and rs7412.^44^ A multimorbidity index (a sum of 15 chronic medical conditions from the Charlson Comorbidity Index^45^ (i.e., diabetes, emphysema, asthma, cancer, osteoarthritis, rheumatoid arthritis, stroke, heart attack, heart failure, heart surgery, angina pectoris, hypertension, peripheral vascular disease, cirrhosis, acquired immune deficiency syndrome [AIDS]) was used as an ordinal variable.^34^ Creatinine was measured from fasting serum samples using Quest Diagnostic spectrophotometry. Fasting began by 9:00 pm the night before testing, and samples were acquired the next morning between 8:00 am and 8:30 am. GFR was calculated using information on study participants’ sex, BMI, ethnicity, and creatine and used as a continuous variable in the analyses.

### 2.6 Statistical analyses

We used generalized estimating equations (GEEs) with an identity link function to analyze the relationship between self‐reported PA at study waves 1 and 3 and blood biomarkers at study wave 3.^46^ GEE was adopted to ensure valid inference because of heteroscedasticity and correlated data (twin pairs). We chose the working exchangeable correlation structure for NfL, Aβ42/40 ratio, and p‐tau217 and the working independent correlation structure for GFAP to yield more power. The results are presented with standardized regression coefficients (βs) and 95% confidence intervals (CIs) with predictor and outcome variables standardized to mean = 0, SD = 1. The distributions of MET hours in study at ages 56 and 67 were skewed and square root transformations of these variables were used in the analyses to improve efficiency. All biomarker variables were log‐transformed, residualized for site and storage time effects, and standardized before analyses. NfL, Aβ42/40 ratio, and GFAP were also winsorized to reduce the effect of outliers (6, 6, and 12 individuals with most extreme values were winsorized, respectively). Study participants who reported > 35 MET hours per day were excluded as outliers (*n* = 2).

We ran three models with self‐reported PA as the predictor variable and AD‐related blood biomarkers as the outcome variable. Model 1 was adjusted for age and race/ethnicity. Model 2 was adjusted for variables in Model 1 and additionally for BMI. Model 3 was adjusted for variables in Model 2 and additionally for socioeconomic status, smoking, alcohol consumption, *APOE* genotype, multimorbidity index, and GFR. We also ran the same models with a binary variable of meeting WHO‐PA recommendation instead of MET hours, and tested for interaction with *APOE* genotype by running the same GEE model with an interaction term for PA and *APOE* genotype. Additionally, to address whether PA–biomarker associations are different according to the intensity level of PA, we ran sensitivity analyses that examined light PA and moderate‐to‐vigorous PA separately. To create the intensity level measures, because a person might engage in many sessions of physical activity during the preceding week varying in intensity, we calculated the amount of MET hours for just their light‐intensity PA and separately the MET hours for just moderate‐to‐vigorous PA. All analyses were carried out in Stata 18.0 (StataCorp LLC).

## 3 RESULTS

The baseline characteristics of the 564 study participants are presented in Table 1. The follow‐up length from age 56 to age 67 was on average 11.4 years (SD 1.4, range 6.1–14.4). The average years of education was 14.1 (SD 2.1, range 8–20). Mean socioeconomic status on the Hollingshead–Redlich index was 43.6 (SD 11.2) corresponding to medium business, minor professional, or technical work. Study participants were on average overweight (mean BMI = 29.2) and a little > 80% did not currently smoke cigarettes. At age 56, the mean amount of self‐reported PA during the preceding week was 2.6 MET hours corresponding to walking slowly for an hour or walking briskly for a half an hour each day. In early late‐life, the mean amount of self‐reported PA during the preceding week was slightly decreased (2.2 MET hours). A total of 56% of the participants fulfilled the WHO‐PA recommendation at age 56 and 43 percent fulfilled it in early late‐life.

**TABLE 1:** Description of the study cohort.

	Characteristics	
	VETSA wave 1 (*n* = 564)	VETSA wave 3 (*n* = 554)
**Age**	55.9 (2.4)	67.3 (2.6)
**Education** (years)	14.1 (2.1)	
**Socioeconomic status**	43.6 (11.2)	
Ethnicity		
Non‐Hispanic	553 (98.1)	
Hispanic	11 (1.9)	
**Body mass index**	29.2 (4.7)	29.8 (5.2)
**Alcohol consumption** (drinks/two weeks)	12.2 (20.8)	13.1 (23.9)
*APOE* genotype		
ε2/ε2	1 (0.2)	
ε2/ε3	75 (13.4)	
ε2/ε4	22 (3.9)	
ε3/ε3	322 (57.6)	
ε3/ε4	127 (22.7)	
ε4/ε4	12 (2.2)	
Smoking status		
Never or former	462 (81.9)	489 (86.9)
Current	102 (18.1)	74 (13.1)
**MET hours**	2.6 (3.9)	2.2 (3.4)
WHO‐PA		
Yes	315 (55.9)	241 (43.3)
No	249 (44.2)	316 (56.7)
Morbidity		
Cardiovascular disease	37 (6.6)	80 (14.4)
Hypertension	203 (36.0)	324 (58.5)
Diabetes	47 (8.4)	142 (25.6)
Stroke	8 (1.4)	29 (5.2)
**Multimorbidity index**	0.9 (1.0)	1.9 (1.5)
**GFR**		76.1 (14.2)
**MCI**	0	59 (10.5)

*Notes*: For continuous and ordinal variables, means and standard deviations are given. For categorical variables, numbers and percentages are presented. Morbidity and multimorbidity items are based on medical history self‐report that a physician told the study participant they had specific conditions. There was some missing information on covariates: 1 missing for wave 3 BMI, wave 3 smoking status; 5 missing for *APOE* genotype; 10 missing for wave 3 MET hours,10 missing for wave 3 diabetes, wave 1 and 3 stroke, wave 1 and 3 hypertension, wave 1 and 3 cardiovascular disease, wave 1 and 3 multimorbidity index, 11 missing for wave 1 diabetes; 23 missing for GFR; and 36 missing for wave 3 alcohol consumption.

Abbreviations: *APOE*, apolipoprotein E; GFR, glomerular filtration rate; MCI, mild cognitive impairment; MET, metabolic equivalent of task; VETSA, Vietnam Era Twin Study of Aging; WHO‐PA, World Health Organization–physical activity (150 minutes of moderate physical activity or 75 minutes of vigorous physical activity or a combination per week).

### 3.1 Prospective analyses: age 56 self‐reported PA and age 67 blood AD‐related biomarkers

Age 56 MET hours was significantly and inversely associated with age 67 NfL in Model 1, Model 2, and in the fully adjusted Model 3 (β = −0.10, 95% CI −0.17 to −0.03; Table 2). More activity was associated with lower NfL. Age 56 MET hours was not significantly associated with age 67 GFAP in Model 1 or Model 2, but it was significantly associated with age 67 GFAP in Model 3 (β = −0.08, 95% CI −0.15 to −0.01). More activity was associated with lower GFAP. Age 56 MET hours was not associated with the age 67 Aβ42/40 ratio or p‐tau217 in Model 1, Model 2, or Model 3 (Table 2).

**TABLE 2:** The association between age 56 MET hours and age 67 blood biomarkers.

Biomarker	Standardized regression coefficient	Confidence interval (lower limit)	Confidence interval (upper limit)
**Model 1**			
p‐tau217	0.01	−0.06	0.08
NfL	**−0.11**	**−0.18**	**−0.04**
Aβ42/40 ratio	−0.01	−0.08	0.07
GFAP	−0.07	−0.14	0.00
**Model 2**			
p‐tau217	0.01	−0.06	0.08
NfL	**−0.11**	**−0.18**	**−0.04**
Aβ42/40 ratio	−0.01	−0.08	0.07
GFAP	−0.07	−0.14	0.00
**Model 3**			
p‐tau217	−0.00	−0.08	0.07
NfL	**−0.10**	**−0.17**	**−0.03**
Aβ42/40 ratio	−0.02	−0.10	0.05
GFAP	**−0.08**	**−0.15**	**−0.01**

*Notes*: Model 1 is age‐ and ethnicity adjusted (*N* = 564). Model 2 is additionally adjusted for BMI (*N* = 564). Model 3 is additionally adjusted for socioeconomic status, smoking, alcohol consumption, *APOE* genotype, multimorbidity index, and GFR (*N* = 546). Statistically significant results are bolded (*P* < 0.05).

Abbreviations: Aβ, amyloid beta; *APOE*, apolipoprotein E; BMI, body mass index; GFAP, glial fibrillary acidic protein; GFR, glomerular filtration rate; MET, metabolic equivalent of task; NfL, neurofilament light chain; p‐tau, phosphorylated tau.

Meeting WHO‐PA recommendation at age 56 was not significantly associated with any of the AD‐related blood biomarkers (Table S1 in supporting information). *APOE* genotype did not significantly modify the association between age 56 MET hours and age 67 blood biomarkers according to our interaction analysis (all *P* values for the interaction terms were > 0.05). In the sensitivity analyses of intensity of PA, higher age 56 moderate‐to‐vigorous PA, but not age 56 light PA, was associated with lower levels of age 67 NfL and GFAP (Tables S3–S4 in supporting information).

### 3.2 Concurrent analyses: age 67 self‐reported PA and age 67 blood AD‐related biomarkers

Age 67 MET hours was significantly and inversely associated with age 67 NfL in Models 1 and 2, but not in Model 3 (β = −0.07, 95% CI: −0.15 to 0.01; Table 3). Age 67 MET hours was not significantly associated with the age 67 Aβ42/40 ratio, p‐tau217, or GFAP (Table 3).

**TABLE 3:** The association between age 67 MET hours and age 67 blood biomarkers.

Biomarker	Standardized regression coefficient	Confidence interval (lower limit)	Confidence interval (upper limit)
**Model 1**			
p‐tau217	0.00	−0.07	0.07
NfL	**−0.08**	**−0.15**	**−0.01**
Aβ42/40 ratio	0.00	−0.07	0.08
GFAP	0.03	−0.04	0.11
**Model 2**			
p‐tau217	0.00	−0.07	0.07
NfL	**−0.10**	**−0.18**	**−0.03**
Aβ42/40 ratio	0.01	−0.07	0.08
GFAP	0.00	−0.07	0.07
**Model 3**			
p‐tau217	0.03	−0.04	0.11
NfL	−0.07	−0.15	0.01
Aβ42/40 ratio	−0.00	−0.08	0.08
GFAP	−0.01	−0.08	0.06

*Notes*: Model 1 is age‐ and ethnicity adjusted (*N* = 554). Model 2 is additionally adjusted for BMI (*N* = 552). Model 3 is additionally adjusted for socioeconomic status, smoking, alcohol consumption, *APOE* genotype, multimorbidity index, and GFR (*N* = 499). Statistically significant results are bolded (*P* < 0.05).

Abbreviations: Aβ, amyloid beta; *APOE*, apolipoprotein E; BMI, body mass index; GFAP, glial fibrillary acidic protein; GFR, glomerular filtration rate; MET, metabolic equivalent of task; NfL, neurofilament light chain; p‐tau, phosphorylated tau.

Meeting the WHO‐PA recommendation at age 67 was positively and significantly associated with NfL in Model 2 (β = −0.17, 95% CI: −0.33 to −0.02) but not in Model 1 or in fully adjusted Model 3 (Table S2 in supporting information). Meeting the WHO‐PA recommendation at age 67 was not significantly associated with age 67 Aβ42/40 ratio, p‐tau217, or GFAP (Table S2). *APOE* genotype did not significantly modify the association between age 67 MET hours and age 67 blood biomarkers according to our interaction analysis (all *P* values for the interaction terms were > 0.05). In the sensitivity analyses of intensity of PA, neither intensity level was significantly associated with blood biomarkers (Table S5–S6 in supporting information).

## 4 DISCUSSION

The present cohort study is among the largest to prospectively and cross‐sectionally examine the association between PA and blood‐based AD‐related biomarkers. We found that higher midlife PA, but not concurrent early late‐life PA, was associated with lower early late‐life NfL and GFAP. NfL has been suggested to be associated with cerebrovascular pathology, white matter hyperintensities, and small vessel burden^47^, ^48^ and even to predict progression of cerebral small vessel disease.^48^, ^49^ Because there was no association between midlife MET hours and early late‐life AD‐specific biomarkers (p‐tau217 and Aβ42/40 ratio), our findings suggest that the inverse association between PA and dementia seen in many studies^1^ may be driven through amyloid‐ and tau‐independent mechanisms that protect against vascular pathology (small vessel disease, white matter hyperintensities). Given the robust evidence on PA's beneficial vascular effects,^50^ this seems logical and is in line with an earlier cross‐sectional study showing an association between higher PA levels and lower NfL levels.^51^ Our finding that more moderate‐to‐vigorous PA, not light PA, may account for the associations between midlife PA and AD‐related blood biomarkers is well in line with earlier studies showing more intense PA to be more favorable for cognitive aging than light PA.^52^, ^53^

Earlier studies on the association between PA and Aβ are highly inconsistent, and no systematic explanation for inconsistent results has been found earlier.^6^, ^18^, ^19^ While most of the earlier evidence on PA and blood‐based Aβ is based on measurements of Aβ40, Aβ42, or Aβ42/40 ratio, studies on the association of PA with newer blood‐based markers of AD pathology (p‐tau181 or p‐tau217) are scarce. One earlier large longitudinal study showed no association between midlife PA and blood‐based Aβ42/40 ratio or p‐tau181 in late life (mean age 76 years)^13^. Another cross‐sectional study showed an association between higher PA levels and lower p‐tau217 levels in cognitively unimpaired and older (≥ 65 years old) South Korean study participants^51^. The association was independent of cerebral Aβ burden (the analyses adjusted for amyloid tracer binding in PET) and may, thus, reflect solely tau pathology. We found no association between MET hours and Aβ42/40 ratio or p‐tau217 levels. This finding suggests no modification of amyloid accumulation by PA. Despite the large emphasis cardiovascular factors have been given in the prevention of dementia,^28^ the neuroimaging markers of AD have often shown little modification by cardiovascular factors.^54^, ^55^

A prevailing notion in how AD develops is that Aβ42 levels decrease peripherally because Aβ42 is deposited in the brain as amyloid plaques.^56^ This notion is supported by many studies showing that lower levels of plasma Aβ42 are associated with greater risk of cognitive decline and AD.^57^ However, 90% of plasma Aβ42 is not derived from the brain but from peripheral activated platelets^58^ due to an innate immune response to inflammation.^59^ In fact, earlier studies have reported that higher (not lower) levels of peripheral Aβ42 predict dementia or cognitive decline in younger, cognitively unimpaired, or preclinical samples.^60^, ^61^ Consequently, a non‐linear or biphasic pattern of blood Aβ42 has been suggested in which initially higher blood Aβ42 levels reflecting higher latent inflammatory state decrease in follow‐up when preclinical AD turns into prodromal or symptomatic AD.^62^, ^63^ In our relatively young sample of US men, the turning point from initially higher blood Aβ42 levels to prodromal decreased Aβ42 levels may be close and may make it difficult to see an association between PA and blood Aβ42/40 ratio. This suggests a biphasic pattern of peripheral Aβ peptides may also be one explanation for diverse results from earlier studies examining the association between PA and blood Aβ peptides.

One of the main hypotheses of how PA acts to prevent dementia has been by decreasing neuroinflammation.^29^ Our results showed an inverse association between midlife PA and early late‐life GFAP (a putative marker of neuroinflammation and astrocytic activation) but not between early late‐life PA and GFAP. The activation of the cells of the immune system (microglia and astrocytes) is not straightforward in the development of AD. When microglia and astrocytes become activated, they may adopt a pro‐ or anti‐inflammatory phenotype,^64^ which is poorly recognized by current biomarkers of AD^65^. Although neuroinflammation is generally thought of as deleterious, it has been suggested that microglial activation especially in early phases of AD may be anti‐inflammatory and protective.^66^, ^67^ Consequently, a non‐linear or curvilinear association has been suggested not only for peripheral Aβ peptides and cognition but also for biomarkers of neuroinflammation and cognition through the AD continuum^68^. This may explain differences in our prospective and cross‐sectional results for PA and GFAP. However, the association between blood‐based biomarkers of neuroinflammation and neuroimaging‐based biomarkers is not straightforward either. For example, GFAP, the blood biomarker of astrocytic activation, is negatively associated with astrogliosis measured with astrocyte tracer ^11^C‐deuterium‐L‐deprenyl (^11^C‐DED) binding in PET.^69^ More research is needed on the patterns of AD‐related biomarkers early in the disease process.

Midlife PA was not associated with early late‐life p‐tau217, a marker of cerebral amyloid and tau pathology^7^. This is contrary to findings from animal studies that show a straightforward decrease in tau accumulation^2^, ^3^. However, most human studies have not found a significant association between PA and tau accumulation^13^, ^18^, ^19^, ^20^, ^22^, ^23^ in line with our study. The only exception is the study^15^ in which cerebral tau burden has been measured in the inferior temporal cortex (Braak stage III) with 18F‐flortaucipir. 18F‐flortaucipir has been shown to be more reliable in advanced stages of AD, Braak V to VI (association cortices in temporal, parietal and occipital cortices and primary sensory and motor cortices).^70^, ^71^, ^72^

Because it has been shown that the associations of PA and many risk factors and diseases is mediated or confounded by BMI, it is important to examine the influence of BMI on the association between PA and AD‐related blood biomarkers. PA is intertwined with bodily mass. Leaner individuals are more likely to stay physically active during adulthood^73^ and physically active individuals may also gain less weight than physically inactive individuals.^74^ BMI is also related to AD‐related blood biomarkers, especially NfL and GFAP, emphasizing the need to take BMI into account while examining the association of PA and AD‐related blood biomarkers.^75^ Higher BMI is associated with lower NfL and GFAP, most likely due to a dilution effect by increased blood volume.^76^ In our study, midlife PA was inversely associated with early late‐life NfL even after BMI was taken into account. This suggests that there is an inverse association between midlife PA and neurodegeneration that is independent of BMI. Future studies that leverage methods such as cross‐lagged panel designs, or genetically informative Mendelian randomization direction‐of‐causation could help disentangle whether PA causally influences NfL.

Our study has limitations. MET hours were zero inflated in this cohort study and reflected PA during the preceding week, which may differ from a study participant's habitual PA level. The number of study participants with zero MET hours at age 56 was 30% and at age 67, it was 47%. Especially our early late‐life PA measure is, thus, limited in detecting differences in very small amounts of PA, which might limit the power of detecting association between PA and blood biomarkers in early late‐life concurrent analyses. PA was self‐reported, which is susceptible to recall bias and over‐ or underestimation. The study population was limited to mainly White, non‐Hispanic men, which limits generalizability to other groups and prevents examination of sex differences in PA–biomarker associations. Because we also did not have information on the biomarker levels from baseline when the study participants were in midlife, we were unable to examine biomarker trajectories. However, AD‐related biomarker levels are likely to be very low (non‐pathological) at average age 56, suggesting that change may not have provided much additional information compared to levels at age 67.

Our study also has several strengths. Our sample is large, and the study design is prospective. The PA variables used in this study are fine grained, taking into account intensity, frequency, and duration of PA. The biomarkers used in this study are well suited to detect early stages of AD, neurodegeneration, and neuroinflammation and correlated well with PET‐based biomarkers.^77^ We were also able to consider kidney function, which has been shown to affect AD‐related blood biomarkers.^78^

## 5 CONCLUSIONS

Evidence to support PA as a significant modifier of amyloid accumulation was not found. However, our study shows that higher midlife MET hours of PA are associated with lower early late‐life NfL and GFAP, which may help to slow progression to dementia by reducing neurodegeneration and neuroinflammation. These associations appear to be accounted for by midlife moderate‐to‐vigorous PA. Better understanding of the mechanisms affecting the accumulation of neuropathology in AD is the only way to effective prevention of AD and is the key to finding efficient therapies for AD. The evidence on the association between PA and in vivo markers specific to AD remains scarce in humans and prospective studies in different ages with fine PA measures and ultrasensitive biomarkers or in vivo imaging are still needed.

## CONFLICT OF INTEREST STATEMENT

The authors declare no conflicts of interest. Author disclosures are available in the Supporting Information.

## CONSENT STATEMENT

Participants gave written informed consent to participate. The study was approved by institutional review boards at University of California, San Diego and Boston University. The study was performed in accordance with the ethical standards as laid down in the 1964 Declaration of Helsinki and its later amendments.

## Supporting information



Supporting Information

Supporting Information
