# Supplementary material for: Prospective study on physical activity and Alzheimer's disease–related biomarkers
Source: Alzheimers Dement. 2026 Aug 19;22(8):e71755. doi: 10.1002/alz.71755 (PMC13490848; doi:10.1002/alz.71755)
Supplement: Supplementary file 2 [file ALZ-22-e71755-s002.docx]

Supplementary Information for ‘Prospective study on physical activity and Alzheimer’s disease–related biomarkers’

Paula Iso-Markku^1,2,3^, Xin M. Tu^1^, Robert A. Rissman, Nathan A. Gillespie^5^, Jeremy A. Elman^1^, Christine Fennema-Notestine^1^, Matthew S. Panizzon^1^, Chandra A. Reynolds^4^, Michael J. Lyons^6^, William S. Kremen^1*^, Carol E. Franz^1*^, for the Vietnam Era Twin Study of Aging

1) Department of Psychiatry, University of California, San Diego, La Jolla, CA, USA; Center for Behavior Genetics of Aging, University of California, San Diego, La Jolla, CA, USA.
2) Institute for Molecular Medicine Finland (FIMM), HiLIFE, University of Helsinki, Helsinki, Finland.
3) Clinical Physiology and Nuclear Medicine, HUS Diagnostic Center, University of Helsinki and Helsinki University Hospital, Helsinki, Finland.
4) Institute for Behavioral Genetics, University of Colorado, Boulder; Department of Psychology & Neuroscience, University of Colorado, Boulder, CO, USA
5) Virginia Institute for Psychiatric and Behavior Genetics, Virginia Commonwealth University, Richmond, VA, USA.
6) Department of Psychology, Boston University, Boston MA, USA

* Drs Kremen and Franz are joint senior authors.

Corresponding author: Paula Iso-Markku

Email: paula.iso-markku@helsinki.fi

This PDF file includes:

Leisure-time physical activity question

Supplementary Tables 1 – 8

**Leisure-time physical activity question**

Please list any sports, fitness or recreational activities in which you participated last week. We are interested only in times that you were physically active. (COMPLETE FOLLOWING CHART. PLEASE DO NOT WRITE IN THE BRACKETED [ ] SPACES.)

Sport, Fitness or Recreation	Times/week	Average minutes per episode

1. ______________________________ [ ] ___________ [ ] ___________ [ ]

2. ______________________________ [ ] ___________ [ ] ___________ [ ]

3. ______________________________ [ ] ___________ [ ] ___________ [ ]

4. ______________________________ [ ] ___________ [ ] ___________ [ ]

5. ______________________________ [ ] ___________ [ ] ___________ [ ]

6. ______________________________ [ ] ___________ [ ] ___________ [ ]

**Supplemental Table 1. The association between age 56 WHO-PA and age 67 blood biomarkers**

| **Biomarker** | **Standardized regression coefficient** | **Confidence interval (lower limit)** | **Confidence interval (upper limit)** |
| --- | --- | --- | --- |
| **Model 1** |  |  |  |
| ptau217 | -0.01 | -0.16 | 0.13 |
| NFL | -0.10 | -0.25 | 0.04 |
| AB42/40 ratio | -0.09 | -0.24 | 0.06 |
| GFAP | -0.09 | -0.23 | 0.05 |
| **Model 2** |  |  |  |
| ptau217 | 0.01 | -0.16 | 0.13 |
| NFL | -0.11 | -0.26 | 0.04 |
| AB42/40 ratio | -0.09 | -0.24 | 0.06 |
| GFAP | -0.10 | -0.24 | 0.03 |
| **Model 3** |  |  |  |
| ptau217 | -0.03 | -0.17 | 0.12 |
| NFL | -0.07 | -0.22 | 0.08 |
| AB42/40 ratio | -0.13 | -0.28 | 0.02 |
| GFAP | -0.11 | -0.25 | 0.03 |

**Model 1** is age- and ethnicity -adjusted (N=564). **Model 2** is additionally adjusted for BMI (N=564). **Model 3** is additionally adjusted for socioeconomic status, smoking, alcohol consumption, *APOE* genotype, multimorbidity index, and GFR (N=546). Statistically significant results are bolded (p < .05). **Abbreviations**: AB42/40 ratio, Amyloid- β 42/40 -ratio; BMI, body mass index; GFAP, Glial Fibrillary Acidic Protein; GFR; Glomerular filtration rate; NFL, neurofilament light; ptau, phosphorylated tau; WHO‐PA, World Health Organization – physical activity (150 minutes of moderate PA or 75 minutes of vigorous PA or a combination per week).

**Supplemental Table 2. The association between age 67 WHO-PA and age 67 blood biomarkers**

| **Biomarker** | **Standardized regression coefficient** | **Confidence interval (lower limit)** | **Confidence interval (upper limit)** |
| --- | --- | --- | --- |
| **Model 1** |  |  |  |
| ptau217 | 0.02 | -0.13 | 0.16 |
| NFL | -0.12 | -0.28 | 0.03 |
| AB42/40 ratio | 0.04 | -0.11 | 0.19 |
| GFAP | 0.11 | -0.03 | 0.26 |
| **Model 2** |  |  |  |
| ptau217 | 0.01 | -0.13 | 0.16 |
| NFL | **-0.17** | **-0.33** | **-0.02** |
| AB42/40 ratio | 0.04 | -0.12 | 0.19 |
| GFAP | 0.05 | -0.09 | 0.20 |
| **Model 3** |  |  |  |
| ptau217 | 0.08 | -0.07 | 0.24 |
| NFL | -0.12 | -0.28 | 0.03 |
| AB42/40 ratio | -0.03 | -0.20 | 0.13 |
| GFAP | 0.02 | -0.13 | 0.17 |
|  |  |  |  |

**Model 1** is age- and ethnicity -adjusted (N=557). **Model 2** is additionally adjusted for BMI (N=555). **Model 3** is additionally adjusted for socioeconomic status, smoking, alcohol consumption, *APOE* genotype, multimorbidity index, and GFR (N=502). Statistically significant results are bolded (p < .05). **Abbreviations**: AB42/40 ratio, Amyloid- β 42/40 -ratio; BMI, body mass index; GFAP, Glial Fibrillary Acidic Protein; GFR, Glomerular filtration rate; NFL, neurofilament light; ptau, phosphorylated tau; WHO‐PA, World Health Organization – physical activity (150 minutes of moderate PA or 75 minutes of vigorous PA or a combination per week).

**Supplemental Table 3. The association between age 56 moderate-to-vigorous MET-hours and age 67 blood biomarkers**

| **Biomarker** | **Standardized regression coefficient** | **Confidence interval (lower limit)** | **Confidence interval (upper limit)** |
| --- | --- | --- | --- |
| **Model 1** |  |  |  |
| ptau-217 | -0.01 | -0.07 | 0.05 |
| NFL | **-0.09** | **-0.16** | **-0.03** |
| AB42/40 ratio | -0.02 | -0.09 | 0.05 |
| GFAP | -0.06 | -0.13 | 0.01 |
| **Model 2** |  |  |  |
| ptau-217 | -0.02 | -0.09 | 0.05 |
| NFL | **-0.10** | **-0.16** | **-0.03** |
| AB42/40 ratio | -0.02 | -0.09 | 0.05 |
| GFAP | -0.07 | -0.13 | -0.00 |
| **Model 3** |  |  |  |
| ptau-217 | -0.02 | -0.09 | 0.05 |
| NFL | **-0.08** | **-0.15** | **-0.02** |
| AB42/40 ratio | -0.03 | -0.10 | 0.04 |
| GFAP | **-0.07** | **-0.14** | **-0.01** |

**Model 1** is age- and ethnicity -adjusted (N=564). **Model 2** is additionally adjusted for BMI (N=564). **Model 3** is additionally adjusted for socioeconomic status, smoking, alcohol consumption, *APOE* genotype, multimorbidity index, and GFR (N=546). Statistically significant results are bolded (p < .05). **Abbreviations**: AB42/40 ratio, Amyloid- β 42/40 -ratio; BMI, body mass index; GFAP, Glial Fibrillary Acidic Protein; GFR, Glomerular filtration rate; MET, Metabolic equivalent of energy expenditure; NFL, neurofilament light; ptau, phosphorylated tau.

**Supplemental Table 4. The association between age 56 light MET-hours and age 67 blood biomarkers**

| **Biomarker** | **Standardized regression coefficient** | **Confidence interval (lower limit)** | **Confidence interval (upper limit)** |
| --- | --- | --- | --- |
| **Model 1** |  |  |  |
| ptau-217 | 0.04 | -0.02 | 0.10 |
| NFL | -0.03 | -0.08 | 0.01 |
| AB42/40 ratio | -0.02 | -0.04 | 0.07 |
| GFAP | -0.02 | -0.07 | 0.03 |
| **Model 2** |  |  |  |
| ptau-217 | 0.04 | -0.02 | 0.09 |
| NFL | -0.03 | -0.08 | 0.02 |
| AB42/40 ratio | 0.02 | -0.04 | 0.07 |
| GFAP | -0.01 | -0.06 | 0.04 |
| **Model 3** |  |  |  |
| ptau-217 | 0.03 | -0.02 | 0.08 |
| NFL | -0.03 | -0.07 | 0.02 |
| AB42/40 ratio | 0.01 | -0.04 | 0.07 |
| GFAP | -0.02 | -0.06 | 0.03 |

**Model 1** is age- and ethnicity -adjusted (N=564). **Model 2** is additionally adjusted for BMI (N=564). **Model 3** is additionally adjusted for socioeconomic status, smoking, alcohol consumption, *APOE* genotype, multimorbidity index, and GFR (N=546). Statistically significant results are bolded (p < .05). **Abbreviations**: AB42/40 ratio, Amyloid- β 42/40 -ratio; BMI, body mass index; GFAP, Glial Fibrillary Acidic Protein; GFR, Glomerular filtration rate; MET, Metabolic equivalent of energy expenditure; NFL, neurofilament light; ptau, phosphorylated tau.

**Supplemental Table 5. The association between age 67 moderate-to-vigorous MET-hours and age 67 blood biomarkers**

| **Biomarker** | **Standardized regression coefficient** | **Confidence interval (lower limit)** | **Confidence interval (upper limit)** |
| --- | --- | --- | --- |
| **Model 1** |  |  |  |
| ptau217 | -0.02 | -0.08 | 0.04 |
| NFL | -0.05 | -0.12 | 0.03 |
| AB42/40 ratio | 0.01 | -0.06 | 0.08 |
| GFAP | **0.07** | **0.00** | **0.13** |
| **Model 2** |  |  |  |
| ptau217 | -0.02 | -0.08 | 0.04 |
| NFL | -0.07 | -0.15 | 0.00 |
| AB42/40 ratio | 0.01 | -0.07 | 0.08 |
| GFAP | 0.04 | -0.02 | 0.10 |
| **Model 3** |  |  |  |
| ptau217 | 0.01 | -0.05 | 0.07 |
| NFL | -0.02 | -0.09 | 0.05 |
| AB42/40 ratio | -0.01 | -0.09 | 0.07 |
| GFAP | 0.04 | -0.02 | 0.11 |
|  |  |  |  |

**Model 1** is age- and ethnicity -adjusted (N=564). **Model 2** is additionally adjusted for BMI (N=562). **Model 3** is additionally adjusted for socioeconomic status, smoking, alcohol consumption, *APOE* genotype, multimorbidity index, and GFR (N=509). Statistically significant results are bolded (p < .05). **Abbreviations**: AB42/40 ratio, Amyloid- β 42/40 -ratio; BMI, body mass index; GFAP, Glial Fibrillary Acidic Protein; GFR, Glomerular filtration rate; MET, Metabolic equivalent of energy expenditure; NFL, neurofilament light; ptau, phosphorylated tau.

**Supplemental Table 6. The association between age 67 light MET-hours and age 67 blood biomarkers**

| **Biomarker** | **Standardized regression coefficient** | **Confidence interval (lower limit)** | **Confidence interval (upper limit)** |
| --- | --- | --- | --- |
| **Model 1** |  |  |  |
| ptau217 | 0.00 | -0.06 | 0.06 |
| NFL | -0.03 | -0.09 | 0.03 |
| AB42/40 ratio | 0.01 | -0.05 | 0.06 |
| GFAP | 0.01 | -0.07 | 0.04 |
| **Model 2** |  |  |  |
| ptau217 | 0.00 | -0.06 | 0.06 |
| NFL | -0.03 | -0.09 | 0.03 |
| AB42/40 ratio | 0.01 | -0.05 | 0.06 |
| GFAP | -0.01 | -0.07 | 0.05 |
| **Model 3** |  |  |  |
| ptau217 | 0.02 | -0.03 | 0.08 |
| NFL | 0.00 | -0.05 | 0.05 |
| AB42/40 ratio | 0.03 | -0.02 | 0.08 |
| GFAP | -0.01 | -0.06 | 0.05 |
|  |  |  |  |

**Model 1** is age- and ethnicity -adjusted (N=564). **Model 2** is additionally adjusted for BMI (N=562). **Model 3** is additionally adjusted for socioeconomic status, smoking, alcohol consumption, *APOE* genotype, multimorbidity index, and GFR (N=509). Statistically significant results are bolded (p < .05). **Abbreviations**: AB42/40 ratio, Amyloid- β 42/40 -ratio; BMI, body mass index; GFAP, Glial Fibrillary Acidic Protein; GFR, Glomerular filtration rate; MET, Metabolic equivalent of energy expenditure; NFL, neurofilament light; ptau, phosphorylated tau.
